# Behavioral and neurobiological mechanisms of punishment: implications for psychiatric disorders

**DOI:** 10.1038/s41386-018-0047-3

**Published:** 2018-03-27

**Authors:** Philip Jean-Richard-Dit-Bressel, Simon Killcross, Gavan P. McNally

**Affiliations:** UNSW Sydney, Sydney, NSW 2052 Australia

## Abstract

Punishment involves learning about the relationship between behavior and its adverse consequences. Punishment is fundamental to reinforcement learning, decision-making and choice, and is disrupted in psychiatric disorders such as addiction, depression, and psychopathy. However, little is known about the brain mechanisms of punishment and much of what is known is derived from study of superficially similar, but fundamentally distinct, forms of aversive learning such as fear conditioning and avoidance learning. Here we outline the unique conditions that support punishment, the contents of its learning, and its behavioral consequences. We consider evidence implicating GABA and monoamine neurotransmitter systems, as well as corticostriatal, amygdala, and dopamine circuits in punishment. We show how maladaptive punishment processes are implicated in addictions, impulse control disorders, psychopathy, anxiety, and depression and argue that a better understanding of the cellular, circuit, and cognitive mechanisms of punishment will make important contributions to next generation therapeutic approaches.

Punishment involves learning about the relationship between behavior and its adverse consequences. It is used in different ways in the contemporary literature. In addiction neuroscience, punishment serves as a tool for assessing persistent drug-seeking in the face of adverse consequences and as a qualitative marker of a compulsive behavioral phenotype underlying individual differences in development of compulsive seeking. In the decision neurosciences, punishment serves as a tool for assessing the influences of risk on decision-making and as a tool for identifying the brain mechanisms of value and choice. In the clinical literature, sensitivity to punishment is assessed across a variety of disorders, including addiction, depression, psychopathy as well as eating disorders, enabling insights into the etiology, maintenance, and treatment of these conditions. It is unsurprising, then, that there is considerable diversity in how punishment experiments are conducted and interpreted. In this article, we consider key theoretical and methodological complexities of punishment, the design choices available, and the implications of these choices for interpretation. We then review some of the brain bases of punishment and psychiatric disorders with perturbations in punishment processing.

## Different forms of aversive learning

**Fig. 1:**
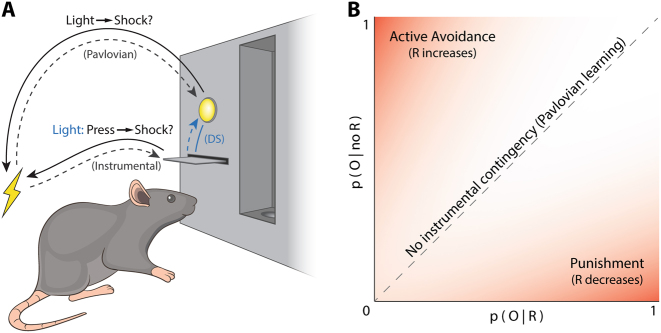
Determinants of aversive associative learning. **a** Even in carefully designed studies (e.g., light → shock Pavlovian fear, or press → shock punishment protocols [solid lines]), aversive events are inevitably embedded within multi-layered contingencies. A shock could be attributed (dashed lines) to behavioral antecedents (e.g., lever-pressing), environmental antecedents (e.g., light), or both. The relative validity of these antecedents determine whether a Pavlovian light → shock, instrumental press → shock, or instrumental discriminative (blue lines) light = [press → shock]) association is formed, in turn determining what behavior is being examined. **b** Contingency space describing relationships between aversive outcome (O) and behaviors (R). Upper left corner of contingency space: O is only likely to occur if the response is not made (response reinforced by contingency). Bottom left corner of contingency space: O is only likely if the response is made (response punished by contingency). If O is independent of responding (dashed line), only Pavlovian learning is likely to occur. DS = discriminative stimulus; O = aversive outcome; R = specified response; p(O|R) = probability of aversive outcome, given the response was made; p(O|no R) = probability of aversive outcome, given the response was not made

Learning about and responding to aversive events is fundamental to survival. The learning and behavior that occurs in response to aversive events depend on the relationships between the aversive event, environmental stimuli and the animal’s behavior (Fig. 1a). In general, we can be passive recipients of aversive events while at other times our actions determine the events we experience. This latter category of response-dependent aversive events can be studied in the laboratory via punishment.

Punishment is instrumental aversive learning. It refers to the suppressive effects of undesirable outcomes on the behaviors that cause them (Table 1). This effect of response-dependent aversive events is symmetrical to the response-promoting effects of reinforcement (instrumental reward learning). Like reinforcement, the instrumental contingency between a response and undesirable outcome causes formation of a response–outcome (R–O) association, i.e., a response–punisher association, that disincentivizes punished responding.

**Table 1:** Characteristics of different aversive learning paradigms

	**Punishment**	**Active avoidance**	**Pavlovian fear**
Contingency	Response → aversive event (response-dependent event)	Response → no aversive event (response-dependent event)	Stimulus → aversive event (response-independent event)
Learning paradigm	Instrumental (punishment)	Instrumental (negative reinforcement)	Pavlovian (fear conditioning)
Associative structure	(DS–)R–O	(DS–)R–[no O]	S–O (CS–US)
Effect on appetitive behavior	Selective reduction in punished response	Displaced by avoidance response	Conditioned suppression
Extinction	Rapid	Slow	Slow

*DS* discriminative stimulus, *O* aversive outcome, *R* response, *S* stimulus

Punishment is closely related to, but distinct from, other forms of aversive instrumental learning—active avoidance and escape learning (Fig. 1b; Table 1). In these, an aversive event is prevented or halted by the performance of an action. They are examples of negative reinforcement, as the specified behavior is *increased* (reinforced) by the contingency between the response and consequence (“negative”, as the consequence of making the response is omission or removal of the stimulus). Active avoidance and punishment are frequently confused, but the key learning processes underpinning them are distinct [1–3]. A helpful way to distinguish these is to note active avoidance involves generation of specific behaviors that avoid or terminate the aversive event (e.g., lever-pressing), while behaviors that allow the aversive event to occur are unspecified and diffuse (grooming, exploring, inactivity, etc.). This results in an R – [no O] association (and possibly a stimulus–response association) that supports responding [4]. Conversely, in punishment the behaviors that avoid the negative event are unspecified and diffuse, while the behavior (e.g., lever-press) that causes the undesirable event is specific.

Punishment is also frequently confused with Pavlovian fear conditioning (Table 1). Indeed, many experiments purporting to study punishment actually involve response-independent delivery of an aversive event regardless of the actions performed. This response-independent contingency is not punishment. Rather, it is the stimulus–outcome (S–O) contingency of Pavlovian fear conditioning. In Pavlovian fear conditioning, a conditioned stimulus (CS) is paired with an aversive stimulus (e.g., an aversive unconditioned stimulus [US]), imbuing the CS with aversive motivational properties. This CS elicits conditioned responses, which can include defensive responses such as freezing but also non-specific suppression of reward behavior (termed conditioned suppression). So, punishment and fear can achieve the same behavioral outcome—suppression of ongoing behavior—via different mechanisms. However, punishment and fear have salient distinguishing characteristics. Punishment suppression is specific to the punished response, whereas Pavlovian fear is not [5, 6]. Punishment causes greater response suppression than fear [7–9]. However, punishment can be more transient than fear; punished behaviors can reappear spontaneously or due to changes in context, and often return rapidly once the punishment contingency is suspended.

## What kinds of events serve as punishers?

A variety of noxious or aversive events serve as punishers when their delivery is made contingent on behavior. The most frequently used punishers are brief footshock, airpuff, or contamination of a palatable solution with quinine (particularly in rodent and non-human primate studies). In humans, the range of punishers is broader and can include the same aversive events (airpuff, loud noises, electrick shock) but also include social exclusion, negative feedback on task performance, and monetary loss. When delivered in a manner contingent on a behavior, these are examples of primary punishers. However, Pavlovian fear CSs can also serve as effective punishers. For example, presentations of a fear CS contingent upon lever-pressing will instrumentally suppress lever-pressing (a procedure known as secondary or conditioned punishment). In these examples, behavior is modified because it causes an adverse event to occur, so these are examples of positive punishment. Still other classes of events can serve as punishers. A reduction or removal of reward, or a stimulus that signals such reductions (e.g., monetary loss, absence of a palatable food), can serve as a punisher when made contingent on a behavior. These kinds of events are frequently used in human neuroimaging or primate single unit recording experiments. These are examples of negative punishment, whereby behavior causes a pleasant stimulus to be removed. It is important to note that reward omission is not always negative punishment. Loss of responding during reward omission can be due to extinction as opposed to response suppression due to punishment. In negative punishment, the rewarding outcome is only omitted if a response is made. In extinction, the response has no bearing on delivery of reward.

One coherent way to treat these diverse events is to suppose that they share a common affective quality: recruitment of an aversive motivational system. This aversive motivational system is able to suppress ongoing reward behavior because it inhibits appetitive motivation. This motivational opponency has proved popular because it provides a short-hand explanation for the common behavioral consequences of otherwise diverse events. There is evidence for common psychological and neurobiological coding of aversive motivational quality [10–13]. However, this equivalence remains poorly understood and there are differences between positive and negative punishment as well as between primary and secondary punishers [14, 15].

An important consideration is the intensity of the aversive event [16]. Low intensity aversive events can be detected but do not cause suppression. Because these events do not result in suppression, they cannot be termed punishers. Such low intensity events can still control behavior, but they do so via different mechanism (see Section 3). Whereas mild to moderate punishers partially suppress behavior, with some observations that this suppression lessens over time (likely due to habituation). Severe punishers result in complete and permanent suppression of responding. A common design choice is to increment punisher intensity across sessions. This allows greater control over rates of suppression, and greater habituation to the punisher, reducing overall suppression [17].

Punisher instensity is also important because it relates to indiviudal differences in punishment. Marchant et al. [18] reported pronounced individual differences in punisment sensitivity, across a large population of rats, when a constant intensity punisher was used. This work revealed a bimodal distribution of punishment sensitive and insensitive rats. Interestingly, when punisher intensity was increased across trials, individual differences were less pronounced. Differences in punisher sensitivity are also important when considering sex differences in punishment. There has been little published work systematically examining sex differences in punishment. However, female rats are more sensitive to footshock than males, and initially show greater response suppression following footshock (see ref. [19]). Interestingly, these sex differences appear to reverse when assessing response suppression after an interval, such that female rats exhibit poorer retention of response suppression. While these differences have important implications for our understanding of punishment processing, these sex differences have not been adequately studied using explicit punishment designs. Moreover, these differences may not extend to other species (e.g., see ref. [20]). The cause of these differences in sensitivity to punishment, between and within individuals, warrant further investigation.

## Learning processes involved in punishment experiments

Typically, the experimenter is interested in the depressive effects of punishment on instrumental responding for reward. That is, the formation and use of an R (lever-press)—O (shock) association. Such associations are formed during punishment [21, 22]. However, other associations are also formed and influence behavior in different ways. Understanding the origin and effects of these other associations is essential.

Consider the simplest punishment experiment. Here a mouse or rat is initially rewarded for lever-pressing (e.g., by a food pellet or intravenous infusion of cocaine). Once responding is established, the lever-presses now also cause delivery of footshock. The mouse will reduce or even cease lever-pressing. This reduction in lever-pressing is the key behavioral dependent variable. However, this reduction in lever-pressing is not sufficient evidence for punishment or use of R–O aversive knowledge. In fact, alone it tells us little about the underlying cause of behavior change.

In any punishment experiment, subjects are influenced by both instrumental and Pavlovian contingencies, even if these are not intended by the experimenter. This is because, in practice, aversive events have both environmental and behavioral antecedents. Moreover, these environmental (Pavlovian) and behavioral (instrumental) antecedents both suppress ongoing behaviors, including lever-pressing for reward. Sometimes these antecedents are well-specified by the experimenter and are known, but in other cases these antecedents are embedded in other features of the experiment and unknown. Whether punishment or fear is controlling behavior in any given punishment experiment is, at least initially, always ambiguous. This is problematic when attempting to attribute effects to one of these processes and not the other and poses significant problems when attempting to understand the effects of brain manipulations. Is lever-pressing reduced because of an instrumental R (lever-press)—O (shock) association? Or does the lever and its spatial location act as a fear CS due to the contingency between the lever and shock, causing Pavlovian fear (S–O) and conditioned suppression?

Whether, when, and how instrumental or Pavlovian associations control behavior in punishment designs has been the subject of significant empirical and theoretical attention [1, 23–25]. Both kinds of association contribute to and cause behavioral suppression in most punishment experiments and various design choices favor one over the other. The contribution of Pavlovian vs. instrumental aversive learning depends on the relative validity of each association. Whether environmental stimuli or the response is a better predictor of shock determines the strength of the associations formed; interposing a stimulus (e.g., a tone) between a response and response-contingent shock can retard the acquisition of instrumental suppression because the validity of the S–O (tone–shock) contingency interferes with (overshadows) the formation of the R–O (press–shock) contingency ([26, 27]).

Pavlovian fear tends to be greatest early in punishment training, and, when using an appropriate experimental design, is reduced across extended training. Fear emerges early due to S–O contingencies between various cues in the apparatus (the chamber, the location of the lever, the sound of lever insertion) and the delivery of the punisher. This fear is weakened across punishment training due to the inevitable extinction of these Pavlovian contingencies if response suppression results in no aversive outcome. Regardless, behavioral suppression early in punishment training often reflects a greater contribution of Pavlovian fear associations than later in training [21, 28].

A similar problem occurs in stimulus control over punishment. Both fear and punishment can be brought under stimulus control. In punishment, such stimulus control is achieved via a discriminative stimulus (DS; traditionally S^D^) that can be used to signal that a punishment contingency is in effect. In fear conditioning a discrete CS is used to signal an impending aversive US. CSs and DSs are superficially similar but not equivalent [29, 30]; punishment DSs and fear CSs differ in terms of what is learned about them and how they control behavior.

One demonstration of these differences is through the use of the blocking procedure [31]. In this procedure, subjects are first trained on one association (e.g., CSA—shock). They then receive compound training of CSA+CSB−shock. When tested for fear to CSB, the subjects show little evidence of having learned fear. CSA is said to have blocked fear learning to CSB. Such blocking is quite general and robust. It shows that what is learned about one CS signaling a shock and a second CS signaling shock is the same because learning about one prevents learning to the other. Blocking is also observed between DSs. For example, what is learned about one DS that signals a period of instrumental punishment is the same as what is learned about a second DS signaling a period of instrumental punishment because they also block learning to each other. However, CSs and DSs do not block learning to each other [29]. The contents of these two different associations are determined by whether the aversive outcome is response-dependent (instrumental) or independent (e.g., Pavlovian).

There are still other associations at work in punishment designs. One relates to direct interactions between the punisher and reinforcer. There are two issues here. The first is motivational or affective interactions between the punisher and reward. These can arise when delivery of the punisher occurs in close temporal proximity to delivery of the reward that sustains responding. This can occur if the same schedule is used for reward and punishment. Under these conditions, there is an unintended contingent relationship between the punisher and the reward. For example, in a typical punishment design using rodents, a shock punisher is invariably delivered immediately via grid floor while a contingently-delivered reward is consumed after due to the requirement of the animal to enter the magazine to consume it or the effects of the intravenous infusion of a drug reward persisting beyond the shock. This unintended signaling relationship, shock → reward, enables a form of learning—counterconditioning—that reduces the aversive value of the shock [32, 33]. The degree of counterconditioning depends on the experimental parameters. In particular, its impact depends on the exact temporal relationships. When the order of events is reversed, so that delivery of reward signals punishment (reward → shock), the effects of punishment are different [23, 34]. The second issue concerns the signaling properties of the punisher itself. Shocks can serve as a DS, signaling whether or not a response will be rewarded [35]. This means that a punisher can suppress instrumental responding for reward, not because the punisher is aversive or noxious and the animal has learned that responding causes shock (i.e., R−O_aversive_), but rather because shock signals that behavior will not be rewarded (i.e., S_shock_ [R−no O_reward_]). Moreover, the reverse is also possible: the presence of the punisher can signal that behavior will be rewarded, thereby *increasing* instrumental responding for the reward (i.e., S_shock_ [R−O_reward_]) [36].

Finally, punishment can be context-specific. When rats are trained to lever-press for reward in one context (context A) then punished for that responding in a second context (context B), responding returns when placed in the original training context (ABA renewal) or in a third context (ABC renewal). Renewal of punished responding has been observed for responding based on food [37] and alcohol [38]. Moreover, opioid self-administration after punishment can be reinstated by priming injections of opioids or benzodiazepines [39, 40]. These effects are reminiscent of the effects of extinction, leading to suggestions that punishment and extinction involve similar contextual learning processes. However, there is only partial overlap between the brain mechanisms of contextual control of punishment and extinction [41, 42] and there are other important behavioral [43] and neurobiological [44] distinctions between them.

## Methodological considerations when studying punishment

Given the complexity of associations formed in punishment experiments, and the fact that many of these associations are not often of primary interest, the literature offers some methodological recommendations. First, it is worth observing and measuring the animal’s behavior in the task. The presence of species-typical defense behavior, such as freezing for rodents, provides one measure of Pavlovian fear that is helpful for interpreting results. Response-punisher associations result in little autonomic disturbance (freezing, piloerection), and are more typically associated with abortive responses [45].

Second, including a different, unpunished behavior in the same task is very useful. For example, in rodent studies this could involve rewarding two responses (e.g., two different levers) and punishing responses on only one. Inclusion of an unpunished behavior serves two purposes. Suppression of responding on the unpunished lever correlates strongly with expression of defensive behaviors such as freezing and reflects Pavlovian fear [5, 6, 28], whereas specific suppression of the punished response is indicative of punishment learning. Different responses on the same manipulandum (lifting vs. pressing a lever; pushing left vs. right on a bar) can be punished vs. unpunished [21]; this approach controls for any fear to the spatial location of the lever. Effects selective to one response reflect use of contingent R–O aversive knowledge whereas effects common to both responses reflect Pavlovian fear or S–O aversive knowledge. The additional purpose of including an unpunished behavior is that the rewarded alternative response supports a more stable suppression of the punished response.

Third, a strong instrumental contingency between a response and punisher supports stronger R–O aversive learning [21]. Ratio schedules produce strong R–O associations [46], so if relatively quick isolation of punishment is desired, ratio schedules are preferable. Even so, ratio schedules still support Pavlovian fear learning at first, thereby affecting interpretation of data from these early sessions. This fear extinguishes in a reasonably continuous fashion while instrumental suppression increases across sessions [21, 28].

Fourth, careful consideration of punishment and reinforcement schedules can avoid many of the interpretative issues associated with direct interactions between the punisher and reward, and use of outcomes as a DS (see above). For example, these direct interactions are more likely to occur when the same schedule of reinforcement is used to deliver the punisher and reward. One way of avoiding such interactions is to deliver rewards on variable interval schedules and the punisher on a fixed ratio schedules [21]. The variable interval schedule for reward encourages relatively stable rates of lever pressing against which to measure punishment suppression and the fixed ratio schedule for punishment encourages strong R–O encoding of punishment. However, many other schedules are possible such as separate variable interval schedules (VI60 v VI90 for reward and punishment; [37]). The important point is to reduce inadvertent signaling relationships between the reward and the punisher.

Fifth, direct comparison of response-contingent vs. non-contingent aversive events in the same experiment can be helpful. This control could be applied in several ways, but one method is via the use of yoking. In between-subjects yoking, stimuli are response-dependent for one subject while yoked (concurrently presented) to another in a response-independent manner. This allows direct comparison of USs/punishers, which are matched in presentation (both number and distribution) but are embedded within differing contingencies. Yoking has inferential limitations [47, 48] but its utility is enhanced in combination with the other suggestions discussed here.

## Brain mechanisms of punishment

Early anxiolytics, particularly barbiturates and benzodiazepines, had such profound “anti-punishment” effects, especially within conflict protocols, that anti-punishment effects were used as a behavioral screen for anxiolytics. The anti-punishment effects of barbiturates and benzodiazepines are well-documented in multiple species at doses that do not affect unpunished behavior (see [49]). They are also observed in conditioned punishment; midazolam abolishes conditioned punishment without affecting unpunished responding or the arousing effects of a footshock [50]. Interestingly, benzodiazepines enhance the acquisition of active avoidance [51, 52], suggesting that benzodiazepine effects on punishment are due to direct actions on instrumental suppression rather than aversive motivation. GABA and benzodiazepine antagonists block the anti-punishment effects of these drugs [53]. Ethanol also has specific anti-punishment effects [54–56] that appear similarly mediated by its action on GABA and the benzodiazepine-binding site [57, 58].

Serotonin (5-HT) is strongly implicated in punishment, with proposed roles in behavioral inhibition [59, 60] and aversive processing [61]. It has been suggested that 5-HT inhibits the reward-coding dopamine system, with 5-HT and dopamine being conceived of as oppositional systems, promoting aversive and appetitive functions respectively [62, 63]. Lesions of serotonin-containing terminals, systemic injections 5-HT antagonists or 5-HT synthesis inhibitors each have anti-punishment effects (see [64]). Acute tryptophan depletion (ATD), a dietary manipulation that putatively impairs 5-HT transmission also appears to reduce aversively-motivated behavior suppression [65].

Drugs that partially agonize 5-HT_1A_ receptors and/or antagonize 5-HT_2_ receptors have strong anti-punishment effects in pigeons [66–68], although these effects are more variable in mammals [69–71]. 5-HT_2C_ agonizts and SSRIs reverse punishment-resistant cocaine-seeking in rats, while serotonin depletion and 5-HT_2C_ antagonism increased punishment-resistant cocaine-seeking [72].

Norepinephrine and norepinephrine agonists also have strong anti-punishment effects [73, 74], and anti-punishment anxiolytics tend to increase norepinephrine activity and release [75, 76]. Concurrently increasing dopamine and norepinephrine transmission, which hypothetically boosts reinforcement and inhibits punishment signals respectively, leads to an increase in disadvantageous choices involving timeout punishments [77]. Endogenous dopamine and norepinephrine may promote reinforcement-sensitivity and punishment-insensitivity, driving the return of responding following omission of an anticipated aversive outcome.

### Forebrain circuits implicated in punishment

**Fig. 2:**
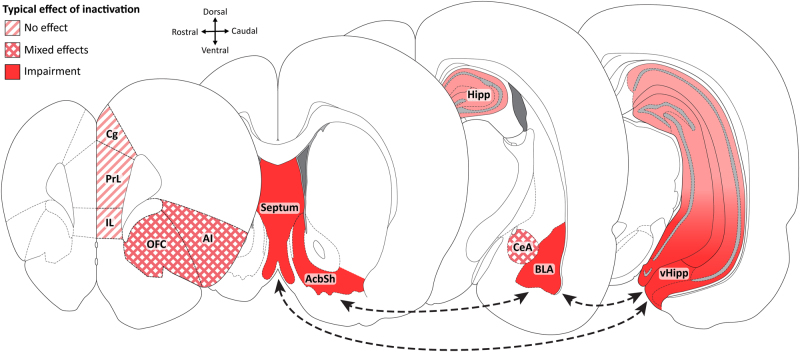
Forebrain regions implicated in punishment. Regions are shaded according to the typical effects of inactivations or lesions of that region on response suppression within punishment protocols. Arrows show regional connectivity implicated in punishment. AcbSh nucleus accumbens shell (ventral striatum), AI anterior insular cortex, BLA basolateral amygdala, CeA central amygdala, Cg cingulate cortex, Hipp hippocampus, IL infralimbic cortex, OFC orbitofrontal cortex, PrL prelimbic cortex, vHipp ventral hippocampus

fMRI studies using a variety of punishment approaches (monetary loss, loss feedback, etc.) implicate human amygdala [78] and its interactions with hippocampus [79] and ventral striatum [80] in punishment (Fig. 2). Anxiolytic, 5-HT, and norepinephrine effects on punishment are linked to amygdala [81–83]. This role is dissociable from Pavlovian fear. In rodents, basolateral amygdala (BLA) lesions and inactivations (particularly caudal portions) attenuate punishment suppression independently from any contributions of Pavlovian fear [28, 50, 84]. The role of central nucleus of the amygdala (CeA) is less clear. Some studies show that CeA mediates Pavlovian but not punishment suppression [85], whereas others suggest a role in punishment of cocaine-seeking [44, 86].

The prefrontal cortex (PFC) has long been implicated in aversion, decision-making and behavioral control [87–89], and has been argued to mediate punishment behavior [90, 91]. We consider the major subdivisions of the rodent PFC, although homologous structures within the human PFC have been similarly implicated in punishment. Within the medial PFC (mPFC), cingulate (Cg) activity is correlated with the magnitude of unpleasantness experienced in response to noxious stimuli [92, 93]. This has been linked to aversion learning [94–96]. Although electrical stimulation of the Cg inhibits behavior [97], lesions of Cg in cats only impair active avoidance, leaving passive avoidance intact [98], suggesting Cg mediates negative reinforcement but not punishment.

The prelimbic cortex (PrL) has been implicated in aversive Pavlovian associations [99], learning appetitive R–O associations, and top-down control of behavior [88, 100]. PrL hypoactivity is associated with impaired sensitivity to punishment in cocaine seeking [101]. Infralimbic (IL) activity is correlated with, sufficient, and necessary for the extinction of instrumental responding [102–105]. This role may extend to suppression of responding due to punishment, fitting with a proposed role for IL in behavioral inhibition [106, 107]. Activity of shock-responsive mPFC neurons is correlated with avoidance of a punished response while stimulation of these shock-responsive neurons results in response suppression [108]. However, PrL or IL inactivations or lesions do not affect primary punishment [109, 110]. Thus, although mPFC activity is correlated with and sufficient for general behavioral suppression, evidence that mPFC is necessary for punishment remains elusive. This remains an important area for further investigation. However, given the key role of medial PFC in Pavlovian fear and the fact that punishment experiments always entail the confounding influence of Pavlovian fear (see above), it will be critical to ensure that appropriate controls for Pavlovian learning are in place to enable correct attribution of a manipulation to effects on punishment as opposed to fear (e.g., inclusion of a second unpunished response, direct assessment of Pavlovian fear, stimulus yoking).

In both rodents and primates, the orbitofrontal cortex (OFC) has been thought to encode value [111, 112], as well as mediate response inhibition and choice [113–116]. The primate OFC contains reward and aversion-coding neurons activated by appetitive and aversive stimuli, respectively [117]. This extends to instrumental tasks and may be topographically partitioned with human medial OFC linked to reinforcement and lateral OFC to punishment [115, 118]. Humans with bilateral OFC lesions are impaired in avoiding disadvantageous options compared to healthy controls in the Iowa Gambling Task [119, 120]. However, in non-human animal studies, OFC inactivation has inconsistent and conflicting effects on punishment, impairing [110], enhancing [121, 122], or having no effect [109] on punishment. These disparities await satisfactory resolution.

The anterior insular (AI) has been strongly implicated in aversion. It is activated in response to, and anticipation of, aversive stimuli [123–127]. This has been linked to pain modulation [128], as well as cognitive and behavioral processes in response to aversive stimuli [91, 129]. AI activity has also been implicated in inhibitory control of behavior [130, 131]. However, AI lesions or inactivations fail to affect punishment suppression [109, 110], but do affect punishment-influenced, subjectively-motivated choice [110, 132].

The ventral striatum, particularly accumbens shell (AcbSh) is also implicated in punishment. Like BLA, AcbSh inactivation increases punished responding [84]. Both AcbSh and BLA may determine overall levels of responding following punishment, decreasing punished responses while increasing safe responses. Kim et al. [108] reported that activity of shock-responsive Acb-projecting mPFC neurons predicted suppression of punished reward-seeking. Stimulation of shock-activated mPFC →Acb neurons also suppressed reward-seeking, suggesting that the mPFC →Acb pathway may mediate punishment suppression, but there is currently no evidence that this projection is necessary for punishment.

Finally, Gray [133, 134] hypothesized that mutually inhibitory behavioral systems compete to guide behavior: the behavioral inhibition (BIS) and behavioral activation (BAS) systems. Punishment activates the BIS, in turn suppressing BAS-driven responding for reward. BIS function was attributed to the septohippocampal system and its monoaminergic afferents from the brainstem [51, 134, 135]. Electrical stimulation of the anterior septum produces somatomotor inhibition [136], while septohippocampal-lesions impair punishment suppression and passive avoidance, independently of spatial learning [137]. Interestingly, septohippocampal lesions often enhanced active avoidance and had less clear effects on Pavlovian fear. Thus, septohippocampal manipulations specifically affect behavioral suppression during punishment, and not aversion generally. These effects mirror those of systemically administered anxiolytics [51, 134, 138], leading to the view that the anti-punishment effects of anxiolytics are driven by disruptions of BIS function [135, 138]. In rats, this role of hippocampus in behavioral suppression appears to be mediated by the ventral, not dorsal, hippocampus [139]

Scales to measure individual differences in human BIS (trait sensitivity to punishment) and BAS (trait sensitvity to reward) have been developed [140]. Higher BIS scores are associated with greater suppression of behaviors in response to negative outcomes [141]. BIS scores are also associated with increased amygdala and hippocampal gray matter volumes [142]. fMRI studies have detected a correlation between BIS scores and punishment-induced amygdala-hippocampus co-activation [79]. Humans with bilateral hippocampus lesions readily switch away from a deck following monetary loss in the Iowa Gambling Task, showing normal physiological responses to punishment (unlike those with amygdala damage), but do not show a preference for advantageous decks (see [143]). This suggests intact aversion processing but impaired use of experienced contingencies to determine choice.

### Midbrain dopamine circuits

**Fig. 3:**
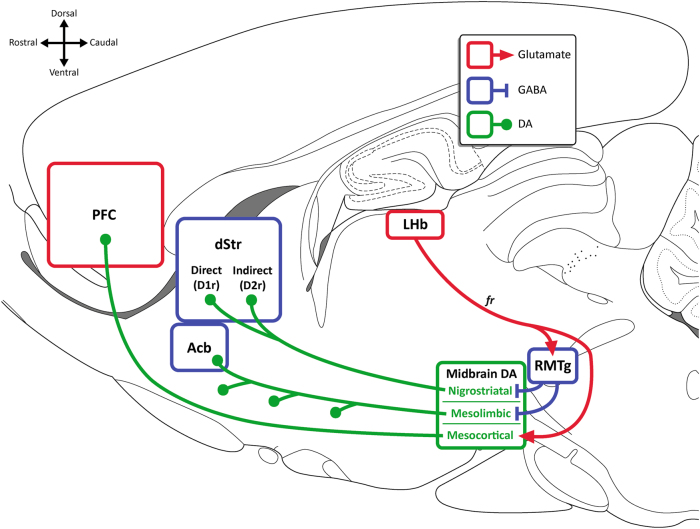
Midbrain dopamine circuits implicated in punishment. Mesolimbic and nigrostriatal projections are generally reward-coding; punishment could be encoded via pauses in DA firing and decreased DA release at projection targets. Mesocortical neurons burst fire to aversive stimuli, ostensibly causing increased DA release within the PFC. Aversion-coding LHb neurons can exert relevant control over each of these pathways, although its role in punishment has been disputed. The role of each of these circuits in punishment remain unclear. Acb nucleus accumbens (ventral striatum), D1r D_1_ receptor, D2r D_2_ receptor, DA dopamine, dStr dorsal striatum, fr fasciculus retroflexus, LHb lateral habenula, PFC prefrontal cortex, RMTg rostromedial tegmental nucleus

Activity of midbrain dopamine (DA) neurons is necessary and sufficient for reinforcement of behavior. Symmetrically, negative outcomes cause pauses in VTA DA firing [11, 12, 144, 145]. It follows that punishment could be due to inhibition of VTA DA neurons. Certainly, inhibiting VTA DA causes conditioned place aversion [146–148] and D_1_ or D_2_ dopamine receptor manipulations in the ventral striatum, a major target of reward-coding VTA DA neurons, can result in place aversion [148, 149]. However, the role of VTA DA in punishment remains unclear (Fig. 3).

VTA DA neurons are inhibited by the lateral habenula (LHb) via the rostromedial mesopontine tegmental nucleus (RMTg) [150, 151]. LHb neurons burst fire to unexpected aversive events and reward omissions [11, 144], and were speculated to mediate punishment learning and behavior [152, 153]. fMRI studies observe habenula BOLD responses to aversive shocks, negative feedback, and omission of anticipated positive feedback [154, 155]. Aversion-coding within LHb may stem from aversion-coding inputs from the globus pallidus (GP; entopeduncular nucleus [EP] in rodents) [156–158] and lateral hypothalamus [159], alongside motivationally-pertinent VTA and 5-HT inputs [160–164]. Optogenetic stimulation of the LHb → RMTg pathway negatively reinforces nose-poking and suppresses acquisition of a reinforced response [152]. However, these studies did not isolate punishment and a variety of approaches to preventing LHb activity do not affect punishment suppression in rats [165]. That said, the LHb appears to play important roles in active avoidance [159] and risky decision-making [166].

Aversive outcomes cause pauses in nigrostriatal DA neuron firing [144, 167]. These neurons have similar inhibitory inputs from the RMTg [168]. Stimulation of the nigrostriatal pathway is rewarding, while inhibition of this pathway can be aversive in real-time place aversion [169]. Increases and decreases in SNc DA firing, and corresponding increases and decreases in striatal DA release, may differentially recruit direct and indirect pathways of the basal ganglia [170–172]. Optogenetic stimulation of D_1_R-expressing medium spiny neurons (MSNs) is reinforcing, while such stimulation of D_2_R-expressing MSNs is punishing [173]. High concentrations of DA preferentially activate D_1_R-expressing direct pathway neurons, while low levels of DA preferentially activate D_2_R-expressing indirect pathway neurons. Thus, Kravitz and Kreitzer [174] suggest LTP of the direct pathway and LTD of the indirect pathway mediates reinforcement, whereas LTP of the indirect pathway and LTD of the direct pathway mediates punishment, in response to bursts and pauses in SNc firing, respectively.

Although this model describes plasticity within striatal pathways that may underpin punishment learning well, it is worth noting that the role of DA in punishment is complex. For example, punishment and reinforcement share common features. Systemic administration of indirect DA agonist amphetamine increases, whereas the DA-receptor antagonist α-flupenthixol decreases, the punishing effect of an aversive CS [85]. This finding is important because it shows that appetitively and aversively motivated conditioned stimuli share common dopaminergic substrates for their influence on instrumental performance. Moreover, some midbrain DA neurons are phasically excited by aversive stimuli (175, 176]). This has been linked to aversion-coding within mesocortical neurons [177–180]. Stimulation of the mesocortical pathway, or its direct excitatory inputs from LHb, is aversive [181], although the role of this circuit in punishment is undetermined.

## Punishment and neuropsychiatric disorders

The study of punishment has great potential to provide insights into decision-making and motivational deficits in neuropsychiatric disorders. To date, most of this research has focused on characterizing the nature of any changes in punishment sensitivity and describing some of the underlying neural correlates. The roles of these alterations in punishment processing, their status as predictors of disorder severity, duration, treatment, and relapse are all poorly understood. Nonetheless, the potential remains to address these deficits and restore normal decision-making to help address the burdens of these disorders.

Risky drug use and an insensitivity to the adverse consequences of drug taking is a diagnostic criterion for substance use disorders [182]. Behavioral addictions, such as gambling disorder, and impulse-control disorders [182], are also characterized by persistent behaviors despite adverse consequences, along with an apparent inability to appropriately suppress that behavior (i.e., impulsivity). Similar deficits have been noted for Obsessive Compulsive Disorder (OCD; [183, 184]) and Attention Deficit Hyperactivity Disorder (ADHD; [185]). These diverse conditions appear to share significant overlap in psychological and neurobiological underpinnings [183, 186]. Thus, punishment and conflict tasks provide important opportunities to probe loss of behavioral control in these disorders. To date, these tasks have been exploited to assess both motivation to seek drug rewards and to assay individual differences in the development of addiction-like behavior, with particular focus on when and how drug-seeking behavior becomes less sensitive to punishment [187–192]. The findings from these tasks have been reviewed in detail elsewhere [193, 194]. One important point to note about these models is that the adverse consequence of drug seeking or taking (i.e., punishment) tends to be immediate (footshock), whereas in human drug users these adverse consequences (e.g., ill health, incarceration) can be delayed. Whether the processes involved in immediate vs. delayed punishment are the same or different remains an important unanswered questions.

The application of punishment to understanding core deficits in neuropsychiatric disorders extends further still. Antisocial personality disorder, conduct disorder and oppositional defiant disorder patients are each characterized by alterations in punishment sensitivity [185, 195, 196]. Individuals with psychopathy or psychopathic traits choose punished options more often than matched controls and do not learn to suppress punished responses across trials [196–198]. This impaired instrumental suppression in psychopathy likely has complex causes but has been attributed, in part, to disrupted prefrontal and amygdala function. Interestingly, both heightened and blunted amygdala activity have been reported in response to aversive stimuli among these populations [199–201] and smaller amygdala volumes reported compared to controls [202, 203]. Moul and colleagues [204] suggest that these differences in amygdala responses are linked to different amygdala subregions, with overactivation linked to alterations in central amygdala valence-coding and underactivation to alterations in BLA encoding of outcome value.

In contrast, depression is associated with increased sensitivity to punishment [205]. Depressed individuals show heightened sensitivity to negative feedback and errors. They also reduce risk-taking more than matched controls following punishment [206]. Depressed individuals can perform comparably or disadvantageously relative to healthy controls on the Iowa Gambling Task (IGT), depending on the task variant used. This profile, possibly reflecting depressed patients’ flattened hyper-sensitivity to punishment and/or hyposensitivity to reward [207–209], is linked to alterations in frontostriatal systems. A key deficit is failing to shift responding when contingencies shift [210] and could be due to a failure to adaptively extinguish avoidance. This is consistent with the observation that depression is associated with increased behavioral inhibition and lower behavioral activation, with lower behavioral activation (which would drive punishment extinction) being the better predictor of continuing depression symptoms [211]. Computational models have linked these deficits to serotonergic dysfunction [212].

## Conclusions

Punishment offers a rich experimental preparation for answering fundamental questions about learning, motivation and decision-making. It also provides unique opportunities to help understand core dysfunctions in complex, neuropsychiatric disorders. We hope it is clear from this primer that many interesting learning processes are involved in even the simplest punishment design, and that determining which of these learning processes are controlling behavior is an important consideration. Remarkably, we are far from a coherent understanding of punishment. We know far less about the brain mechanisms of punishment than those for reinforcement or for other forms of aversive learning. Although some key brain regions have been identified, the precise nature of these contributions to learning and motivational processes, relevant connectivity, and cell types, remain poorly understood and await detailed investigation.
